# Effect of vaccine reminder and tracker bracelets on routine childhood immunization coverage and timeliness in urban Pakistan (2017-18): a randomized controlled trial

**DOI:** 10.1186/s12889-020-09088-4

**Published:** 2020-07-11

**Authors:** Danya Arif Siddiqi, Rozina Feroz Ali, Mehr Munir, Mubarak Taighoon Shah, Aamir Javed Khan, Subhash Chandir

**Affiliations:** 1 IRD Global, 15 Beach Road #02-01, Singapore, 189677 Singapore; 2 IRD Pakistan, 4th Floor Woodcraft Building, Korangi Creek, Karachi, 75190 Pakistan; 3 Department of Global Health and Social Medicine, Harvard Medical School, 641 Huntington Avenue, Boston, MA 02115 USA

**Keywords:** Immunization coverage and timeliness, Silicone bracelets, Immunization reminders

## Abstract

**Background:**

Inability to track children’s vaccination history coupled with parents’ lack of awareness of vaccination due dates compounds the problem of low immunization coverage and timeliness in developing countries. We evaluated the impact of two types of silicone immunization reminder bracelets for children in improving immunization coverage and timeliness of Pentavalent-3 and the Measles-1 vaccines.

**Methods:**

Children < 3 months were enrolled in either of the 2 intervention groups (Alma Sana Bracelet Group and Star Bracelet Group) or the Control group. Children in the intervention groups were provided the two different bracelets at the time of recruitment. Each time the child visited the immunization center, a hole was perforated in the silicone bracelet to denote vaccine administration. Each child was followed up till administration of Measles-1 vaccine or till 12 months of age (if they did not come to the center for vaccination). Data was analyzed using the intention-to-treat population between groups. The unadjusted and adjusted Risk Ratios (RR) and 95% confidence interval (CI) for Pentavalent-3 and Measles-1 coverage at 12 months of age were estimated through bivariate and multivariate analysis. Time-to-Pentavalent-3 and Measles-1 immunization curves were calculated using the Kaplan–Meier method.

**Results:**

A total of 1,445 children were enrolled in the study between July 19, 2017 and October 10, 2017. Baseline characteristics among the three groups were similar. Up-to-date coverage for the Pentavalent-3 /Measles-1 vaccine at 12 months of age was 84.6%/72.0%, 85.4%/70.5% and 83.0%/68.5% in Alma Sana Bracelet group, Star Bracelet group and Control group respectively but the differences were not statistically significant. In the multivariate analysis, neither the Alma Sana bracelet (adjusted RR = 1.01; 95% CI: 0.96-1.06), (adjusted RR: 1.05; 95% CI: 0.97-1.13) nor the Star bracelet (adjusted RR = 1.01; 95% CI: 0.96-1.06) (adjusted RR: 1.03; 95% CI: 0.95-1.11) was significantly associated with Pentavalent-3 vaccination or Measles-1 vaccination.

**Conclusion:**

Although we did not observe any significant impact of the bracelets on improved immunization coverage and timeliness, our findings add to the existing literature on innovative, low cost reminders for health and make several suggestions for enhancing practical implementation of these tools.

**Trial registration:**

ClinicalTrials.gov NCT03310762. Retrospectively Registered on October 16, 2017.

## Background

Routine immunization constitutes one of the most powerful and universally cost-effective interventions in public health, with Gavi Alliance -supported countries estimated to have prevented approximately 1.7 million future deaths in 2018 as a result of vaccination [1]. Major investments in immunization across the world led to 85% global coverage of the Diphtheria-tetanus-pertussis (DTP-3) vaccine in 2017, up from 72% in 2000 [2]. Moreover, in the same period, the number of children who missed out on basic vaccines in Gavi Alliance-supported countries was nearly halved [3]. Despite these achievements, however, many countries still struggle with low immunization coverage and timeliness, particularly in resource-poor regions of the world. In Pakistan, only 66% of all under-2 children receive all basic vaccines, and only 51% of these are age-appropriate [4]. Low immunization coverage not only exposes individual children to the risk of illness, disability, and death but also decreases herd immunity [5], an essential component of national disease prevention strategies. While low coverage is detrimental, delays in immunization are an additional problem, as the temporal spacing of vaccines is designed to maximize immunity, and deviance from the schedule dampens vaccine efficacy (even if all doses are eventually received) [6, 7]. There is thus a dire need to boost both immunization coverage as well as timeliness in countries such as Pakistan, where rates of vaccine schedule compliance are below optimal levels.

In recent years, there has been extensive evidence within public health literature indicating that suboptimal immunization coverage and timeliness is attributable not only to supply-side deficiencies, but also to demand-side problems such as caregiver complacency, forgetfulness, and unawareness of required number and timing of doses [8]. Among the main reasons for under-utilization of immunization services by caregivers is the inability to understand the vaccination schedule, and to remember the due dates for subsequent immunization visits [9]. Demand-side barriers are therefore recognized as equally detrimental to national immunization programs as the pervasive supply-side problems.

Globally, one of the most widely used methods to communicate the vaccination schedule to caregivers is the paper-based immunization card. However, immunization cards are associated with several problems, including lack of durability, potential misplacement, and failure to empower caregivers who cannot read [10, 11]. Studies have confirmed that cardholder prevalence is low in the most resource-poor environments [10], and that card under-utilization is a widespread problem. Mobile-technology based reminder-recall (R/R) interventions have tried to address this issue, but their success has also been partial [12]. Like immunization cards, which require literacy, interventions requiring mobile-phone ownership fail to penetrate the lowest socio-economic strata as it is difficult to implement them in the poorest countries, where infrastructure for technological interventions is absent or subpar, thus widening the global immunization equity gap [13]. Apart from immunization cards and mobile-technology based interventions, Iin Pakistan, one of the strategies deployed in response to caregivers’ failure to visit immunization centers on time is the implementation of door-to-door vaccination and awareness campaigns by the government. Such campaigns, however, neither optimize resource allocation, nor complement routine immunization activities and cannot be a long term sustained strategy for improving coverage.

There is thus a programmatic and research gap regarding strategies to improve immunization coverage and timeliness in low-literacy communities through cost-effective, sustainable solutions that can easily be integrated into the existing public health system and focus on transforming caregivers from passive to active recipients of immunization services.

We aimed to evaluate the effect of silicone bracelets in improving immunization coverage of Pentavalent-3 and the Measles-1 vaccines in children under 2 years, in a low-literacy, peri-urban community. The secondary outcome focused on caregiver feedback regarding the bracelet including its value, ease of use, and visibility.

## Methods

### Study design and participants

We conducted a multicenter, three-arm parallel group, randomized control trial undertaken at four immunization centers in Karachi, Pakistan. The study was conducted in Landhi Town which constitutes one of the largest peri-urban towns in the south of Karachi city, in Pakistan’s Sindh province with an estimated population of around one million and an annual birth cohort of 41,000 children in 2017-18. Administratively, Landhi Town is subdivided into 12 Union Councils (UCs), 5 of which are primarily Pashtun dominated while the rest are represented by Urdu speaking, Punjabi and Sindhi ethnicities with income levels ranging from lower middle income to low-income communities. The town also contains a large industrial zone with a substantial proportion of the workforce employed as factory workers and migration being a distinctive feature within the overall population.

Health care provision in Landhi Town falls under a network of both public and private health care providers. Specifically, immunizations are provided by government-run Expanded Programme on Immunization (EPI) centers consisting of a network of 29 vaccinators who administer vaccines. Out of a total 17 EPI centers in the Town, we selected four contiguously located high volume centers, whose catchment areas included seven out of 12 union councils in Landhi Town. As per the Pakistan Demographic and Health Survey (2017-18) [4], 82% of all 12-23 month old children in Sindh province had received the (BCG; Bacille Calmette Guérin) vaccination. The coverage for Pentavalent-3 and Measles-1 vaccine was 59% and 61% respectively.

The inclusion criteria for the study included children presenting to any of the four selected immunization centers for BCG or Pentavalent-1 vaccination, accompanied by a primary caregiver, healthy, and had been residents of the catchment area for more than six months. Exclusion criteria included children older than 3 months of age or their caregivers planning to visit a non-study immunization center for the follow-up immunizations. Written informed consent was obtained from the parents or caregivers of all children.

The study was approved by the Committee on the Use of Human Subjects at Harvard University, USA and the Institutional Review Board of Interactive Research and Development, Pakistan. The trial was registered with ClinicalTrials.gov, number NCT03310762.

### EPI vaccination schedule

Pakistan’s routine immunization schedule in 2018 included BCG (Bacille Calmette-Guérin) vaccine at birth, three doses of pentavalent (DPT, HepB, Hib) vaccine, three doses of pneumococcal conjugate vaccine (PCV) and three doses of oral polio vaccine at 6, 10 and/or 14 weeks of age, and two doses of measles vaccine at 9 and 15 months of age.

### Procedures

Among the caregiver-child pairs visiting the centers, eligible children identified through the screening process were approached by our trained field staff for obtaining written consent (from their caregivers). Those who consented to participate were enrolled in the study, randomized and information was collected about the child’s current and past vaccination as well as demographic characteristics and socio-economic status. Depending upon the child’s allocated group, the child was provided the intervention (detailed below). After that, data was collected on the child’s vaccination status each time the child visited the immunization center, and if the child was in the treatment group, the relevant intervention procedures were followed (detailed below). As per the recommended EPI schedule, each child was due to visit the center 3-4 times (depending on enrollment vaccine) up till the final vaccine visit for the study at Measles-1 vaccine. At the Measles-1 vaccine visit, a completion form was administered to collect data on experiences of using the bracelets as well as self-reported compliance of wearing the bracelet.

For children who did not visit the center for the recommended number of visits by 12 months of age, we conducted phone calls to document the child’s immunization status. The primary caregiver who had brought the child for immunization was asked to refer to the child’s immunization card to determine the vaccines given and their dates. In case the immunization card was lost, the caregiver could not read the immunization card or the caregiver’s phone number was not available, a household visit was done to document the child’s immunization history. During the household visit, the immunization status was determined through the official EPI card and if this was not available, a verbal recall for the immunization history was taken. A schematic representation of the study procedures is included in the Supplementary Figure 1.

### Intervention

**Fig. 1:**
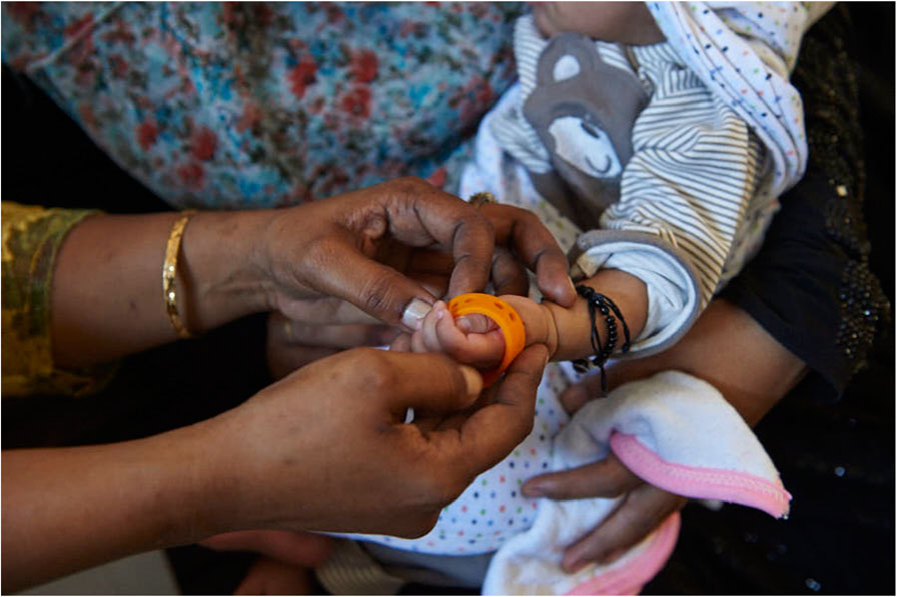
Picture from the field of a child wearing an adapted version of Alma Sana Bracelet

Our two intervention groups comprised of two different types of immunization reminder bracelets. Intervention Group A was provided with a bracelet developed by Alma Sana Inc., a 501(c)3 non-profit organization founded in Indianapolis, Indiana, US. Following a short formative phase, the bracelet was adapted to the Pakistani context through feedback from mothers and vaccinators and involved changes in the color, choice of symbols and denotation of the child’s age on the bracelet, as well as adapting it to suit Pakistan’s EPI schedule. The final adapted bracelet had the recommended age of the child denoted in weeks/months for receiving the vaccine followed by symbols representing each of the vaccines due at that age (Fig. 1).

Each time the child came for vaccination with the bracelet, our study staff perforated a hole in the particular symbol denoting the vaccine that the child had received on that visit and explained to the caregivers the number of vaccines the child still had to receive to complete the routine immunization schedule. Caregivers could, therefore, look at the bracelet and know which vaccines the child had already received and the recommended age of the future visits. The bracelets were manufactured in two different sizes to ensure they fit the child’s wrist as he/she grew older.

**Fig. 2:**
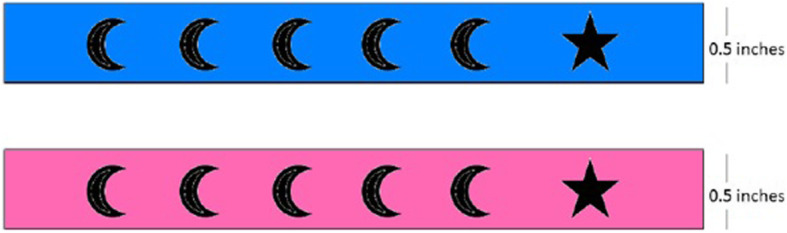
Star Bracelet

Intervention Group B was provided with a simple silicone bracelet “Star Bracelet” that consisted of six symbols (five crescents and one star) denoting the sixvisits that the child is supposed to make to the immunization clinic to complete the routine immunization schedule. The bracelet was designed with the rationale to motivate parents to make all six visits to the immunization center in order to reach the ‘star’ symbol on the bracelet (Fig. 2). Similar to Intervention Group A, each time the child visited the center with the bracelet, the study staff punched a hole to denote the child’s visit to the immunization center and explained to the caregiver to complete all six immunization visits to reach the star symbol on the bracelet. The bracelets were manufactured in two different sizes and colours (pink for girls and blue for boys).

As explained above, although the rationale behind the two different types of bracelets and their mode of use was the same, the bracelets differed in aspect of their designs. The adapted Alma Sana bracelet presented the entire vaccination schedule, along with the different antigens and their stipulated times of administration. The Star bracelet on the other hand had a simpler design, only denoting the six immunization visits (without specifications of the antigens and the timings of the visits). A simpler design provided information in an easily comprehensible way, specifically for mothers with low literacy levels who cannot read and understand the complicated EPI schedules. Through using these two different types bracelets, we can evaluate if there is any difference in vaccination outcomes as a result of conveying varying degrees of information, in different formats to caregivers regarding their child’s immunization schedule.

The control group participants received the standard of care which included receiving the routine EPI vaccinations as per Pakistan’s EPI Immunization schedule and the vaccinator recording the child’s immunization data in the EPI immunization card provided to the caregivers. The difference between the intervention and standard care was only the provision of bracelets and no extra counselling/information was provided to the caregivers.

### Sample size

We expected a 60% coverage rate of Pentavalent-3/ vaccine and a 50% coverage rate of Measles-1 vaccine in the control group, and hypothesized an increase in coverage by an absolute number of 10% in the Pentavalent-3 vaccine coverage (from 60 to 70%) or Measles-1 vaccine coverage (from 50 to 60%). With 80% power (and a 2-sided type 1 error of 5%), we estimated a sample of 1,062 participants (354 in each group) to detect a difference of 10% between intervention and control group proportion for Pentavalent-3 vaccine coverage rate, and a sample size of 1,155 participants (385 in each group) to detect a difference of 10% between intervention and control group proportion for Measles-1 vaccine coverage rate. On the basis of these numbers, a sample size of 1,155 participants was needed to achieve at least 80% power to realize both objectives. Accounting for a potential dropout rate of 20% during the follow-up period, our final sample size was 1,446 infants (482 in each study group).

### Randomization

Participants were randomly assigned (1:1:1) to either the Intervention Group A (Alma Sana Bracelet). Intervention Group B (Star Bracelet) or control group. The randomization sequence was generated in Stata version 13 using random block sizes of 3, 6, 9 and 12. After confirming the eligibility criteria, the next available randomization number in the immunization center (in chronological order) was assigned. The allocation sequence was concealed from the study staff responsible for screening and enrolling participants in sequentially numbered, opaque, sealed envelopes and was only revealed post-randomization. The statistical analysis plan was developed prior to start of the study.

### Measures

Our study outcome of interest included the coverage and timeliness of Pentavalent-3 and Measles-1 vaccines at 12 months of age in the intervention versus control groups. We also investigated the self-reported compliance of caregivers on the child actually wearing the bracelet along with some general feedback on the bracelet itself.

### Analysis

All study data were collected on paper based forms and transferred to a secure electronic database on a daily basis. The data were analyzed using STATA version 15 (StataCorp. 2017. Stata Statistical Software: Release 15. College Station, TX: StataCorp LLC).

For baseline characteristics, we used frequencies (%) for categorical data, means, and standard deviation (SD) for continuous data. The baseline characteristics were compared by using the Student’s t-test for continuous variables and the Pearson’s chi-square test for categorical variables. We analyzed data using the intention-to-treat population between groups. The unadjusted and adjusted Risk Ratios (RR) and 95% confidence interval (CI) for Pentavalent-3 and Measles-1 coverage at 12 months of age were estimated through bivariate and multivariate analysis. Two-sided *P* values were reported, and the values of ≤.05 were considered statistically significant. The variable selection was performed using a stepwise forward model (*p* < 0·10) for each of our two outcomes (Pentavalent-3 and Measles-1) separately. Time-to-Pentavalent-3 and Measles-1 immunization curves were calculated using the Kaplan–Meier (KM) method. The intervention and control groups were compared for effect on timely completion of Pentavalent-3 and Measles-1 immunization using the log-rank test.

We also assessed the Pentavalent-3 and Measles-1 coverage at 12 months of age among the 3 allocation groups and performed chi-squared tests to determine the effect of the treatment on coverage. Additionally, caregiver feedback in the two intervention groups regarding their overall satisfaction with the bracelets and visibility of bracelet was assessed through data collected in the completion form. Caregiver satisfaction was measured through feedback on the utility of the bracelets, its ease of use and whether caregivers would recommend it to others. Visibility of the bracelet was assessed by enquiring the self-reported compliance of children wearing the bracelet and where it was kept when the child was not wearing it.

## Results

**Fig. 3:**
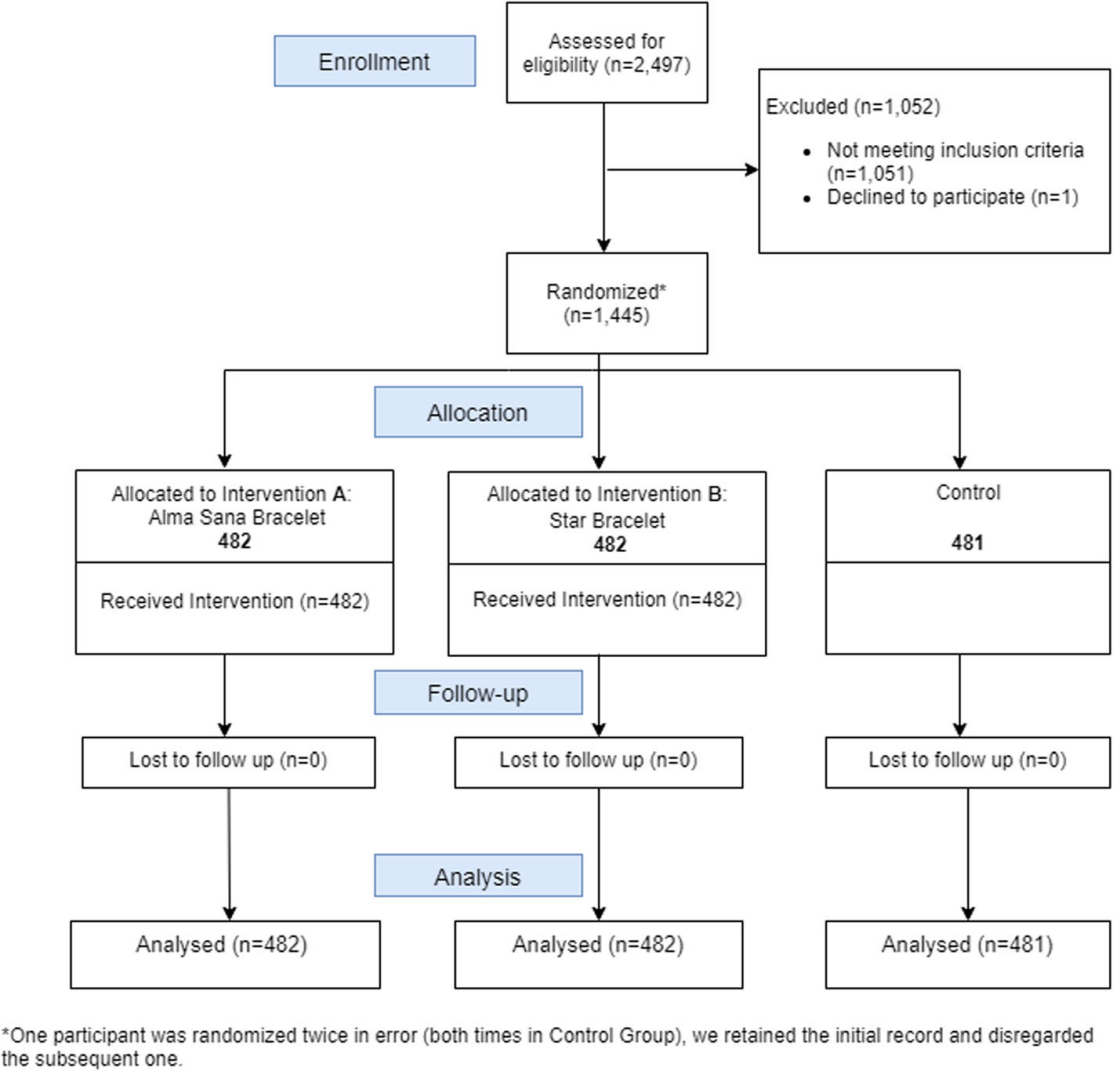
Study Participant Flow from July 19, 2017 until October 16, 2018 for all enrolled children across the four study sites in Landhi Town, Karachi

From July 19, 2017 to October 10, 2017, 1,445 children were enrolled and correctly followed until 12 months of age (until October 16, 2018) (Fig. 3).

The baseline characteristics of study participants in both groups at the time of enrolment were similar for the majority of the variables. We observe some differences across the father’s education level in groups A and B compared to the control group, the secondary education level (6-10 years) was approximately 7% higher in groups A and B compared to the control group. Participant’s in groups A and B had a higher proportion with father’s occupied in ‘Other’ occupation category compared to the control group. We also observe mother’s with more antenatal visits in group B compared to the control group (Table 1). In the entire cohort, a little over half of the study participants were of Urdu speaking ethnicity with fathers having an average of 8.7 years of education (SD: 4.4) and mothers having an average of 8.1 years of education (SD: 4.5). Almost half of the fathers were employed as either skilled laborers (27.9%) or in private jobs (20.0%) whereas almost all of the mothers in the sample were housewives (99.2%). The average monthly household income was USD 175 and there were on average 8 members per family (SD = 4.8). Almost all children in the study (91.0%) were born at a health facility with the delivery assisted by a doctor (88.8%).

**Table 1:** Baseline characteristics of study participants by allocation group collected from July 19, 2017 until October 10, 2017, across all 4 study sites in Landhi Town, Karachi

	Intervention A: Adapted Alma Sana Bracelet (n = 482)	Intervention B: Star Bracelet (n = 482)	Control (n = 481)	Total (n = 1445)
Age at BCG (in weeks), m (SD)	2.6 ± (2.4)	2.6 ± (2.6)	2.4 ± (2.2)	2.5 ± (2.4)
Enrolment Vaccine, n(%)				
BCG	217 (45.0)	200 (41.5)	216 (44.9)	633 (43.8)
Penta-1	265 (55.0)	282 (58.5)	265 (55.1)	812 (56.2)
Female Child, n(%)	257 (53.3)	237 (49.2)	243 (50.5)	737 (51.0)
Age of Mother (in years), m (SD)	26.7 ± (4.7)	26.5 ± (4.6)	26.5 ± (4.7)	26.6 ± (4.6)
Age of Father (in years), m (SD)	31.8 ± (5.6)	31.6 ± (5.6)	31.9 ± (5.8)	31.8 ± (5.7)
Education of Father (in years), n(%)				
0 years	65 (13.5)	57 (11.8)****	87 (18.1)	209 (14.5)
1 - 5 years	28 (5.8)	37 (7.7)	35 (7.3)	100 (6.9)
6 - 10 years	255 (52.9)***	251 (52.1)*	218 (45.3)	724 (50.1)
11 – 12 years	96 (19.9)	77 (16.0)	83 (17.3)	256 (17.7)
≥ 13 years	38 (7.9)**	60 (12.4)	58 (12.0)	156 (10.8)
Mean years, m (SD)	8.8 ± (4.2)	8.9 ± (4.2)	8.4 ± (4.7)	8.7 + (4.4)
Education of Mother (in years), n(%)				
0 years	86 (17.8)	76 (15.8)	84 (17.5)	246 (17.0)
1 - 5 years	56 (11.6)	49 (10.2)	56 (11.6)	161 (11.1)
6 - 10 years	221 (45.9)	233 (48.3)	219 (45.5)	673 (46.6)
11 - 12 years	79 (16.4)	83 (17.2)	73 (15.2)	235 (16.3)
≥ 13 years	40 (8.3)	41 (8.5)	49 (10.2)	130 (9.0)
Mean years, m (SD)	7.9 ± (4.5)	8.3 ± (4.4)	8.1 ± (4.5)	8.1 ± (4.5)
Father’s Occupation, n(%)				
Unskilled	74 (15.4)	81 (16.8)	90 (18.7)	245 (17.0)
Skilled Labor	124 (25.7)	144 (29.8)	135 (28.1)	403 (27.9)
Retail/Sales job	84 (17.4)	88 (18.3)	71 (14.8)	243 (16.8)
Private job	104 (21.9)	88 (18.3)	97 (20.2)	289 (20.0)
Other	96 (19.9)	81 (16.8)	88 (18.3)	265 (18.3)
Mother’s occupation, n(%)				
Housewife	477 (99.0)	481 (99.8)	476 (99.0)	1434 (99.2)
Other	5 (1.0)	1 (0.2)	5 (1.0)	11 (0.8)
Ethnicity, n(%)				
Urdu	289 (60.0)	276 (57.3)	293 (60.9)	858 (59.4)
Pashto	80 (16.6)	76 (15.8)	86 (17.9)	242 (16.8)
Hindko	51 (10.6)	57 (11.8)	46 (9.6)	154 (10.7)
Punjabi	20 (4.2)	28 (5.8)	30 (6.2)	78 (5.4)
Sindhi	10 (2.1)	10 (2.1)	8 (1.7)	28 (1.9)
Other	32 (6.6)*	35 (7.3)***	18 (3.7)	85 (5.9)
Household Income per month (USD), m (SD)	176.8 ± (106)	175.3 ± (78)	173.0 ± (102)	175.0 ± (96.0)
Number of family members, m (SD)	8.0 ± (4.4)	8.2 ± (5.0)	8.3 ± (4.9)	8.2 ± (4.8)
Number of live births by child’s mother, m (SD)	2.5 ± (1.6)	2.5 ± (1.4)	2.6 ± (1.5)	2.6 ± (1.5)
Number of ANC visits by mother during last pregnancy, m (SD)	7.2 ± (3.2)	7.6 ± (3.2)***	7.1 ± (3.0)	7.3 ± (3.1)
Place of delivery of child, n(%)				
Health Facility	440 (91.3)	431 (89.4)	444 (92.3)	1315 (91.0)
Home	42 (8.7)	51 (10.6)	37 (7.7)	130 (9.0)
Who delivered child, n(%)				
Doctor	430 (89.2)	420 (87.1)	433 (90.0)	1283 (88.8)
Midwife	34 (7.1)	47 (9.8)	33 (6.9)	114 (7.9)
Nurse	16 (3.3)	14 (2.9)	12 (2.5)	42 (2.9)
Other	2 (0.4)	1 (0.2)	3 (0.6)	6 (0.4)
Child has been breastfed (Yes), n(%)	457 (94.8)	453 (94.0)	455 (94.6)	1365 (94.5)

*p < 0.05; **p < 0.04; ***p < 0.02; **** *p* < 0.01

*BCG* Bacille Calmette Guérin, *SD* Standard Deviation, *m* mean, *ANC* Antenatal Care, *USD* US Dollar

### Immunization coverage and timeliness

Up-to-date coverage for the Pentavalent-3 vaccine at 12 months of age was 84.6% (408/482), 85.4% (411/481) and 83.0% (399/481) in groups A (Alma Sana Bracelet), B (Star Bracelet) and C (Control) respectively (Table 2). The differences in coverage rates were not statistically significant compared to the control group or between the two intervention groups (*p* > 0.05). Up to date Measles-1 coverage at 12 months of age was slightly higher in the group A (Alma Sana Bracelet) (72.0%, 345/479) and group B (Star Bracelet) (70.5%, 339/481) as compared to the control group (68.5%, 329/480), but the differences were not statistically significant (p > 0.05, not shown).

**Table 2:** Up-to-date antigen wise coverage at 12 months of age in study participants by allocation group

	Intervention A: Adapted Alma Sana Bracelet		Intervention B: Star Bracelet		Control	
Total Number of Children, n (%)	482		482		481	
At BCG	217/482	(45.0)	200/482	(41.5)	216/481	(44.9)
At Pentavalent-1	265/482	(55.0)	282/482	(58.5)	265/481	(55.1)
Pentavalent-1*	208/217	(95.8)	183/200	(91.5)	202/216	(93.5)
Pentavalent-2	445/482	(92.3)	434/482	(90.0)	432/481	(89.6)
Pentavalent-3	408/482	(84.6)	411/481	(85.4)	399/481	(83.0)
Measles-1	345/479	(72.0)	339/481	(70.5)	329/480	(68.5)

*For children enrolled at *BCG*; Bacille Calmette Guérin

** None of the differences are significant at p ≤ 0.05

For Pentavalent-3 and Measles-1 analysis, we have excluded the children who passed away before they were due for the relevant vaccine

**Fig. 4:**
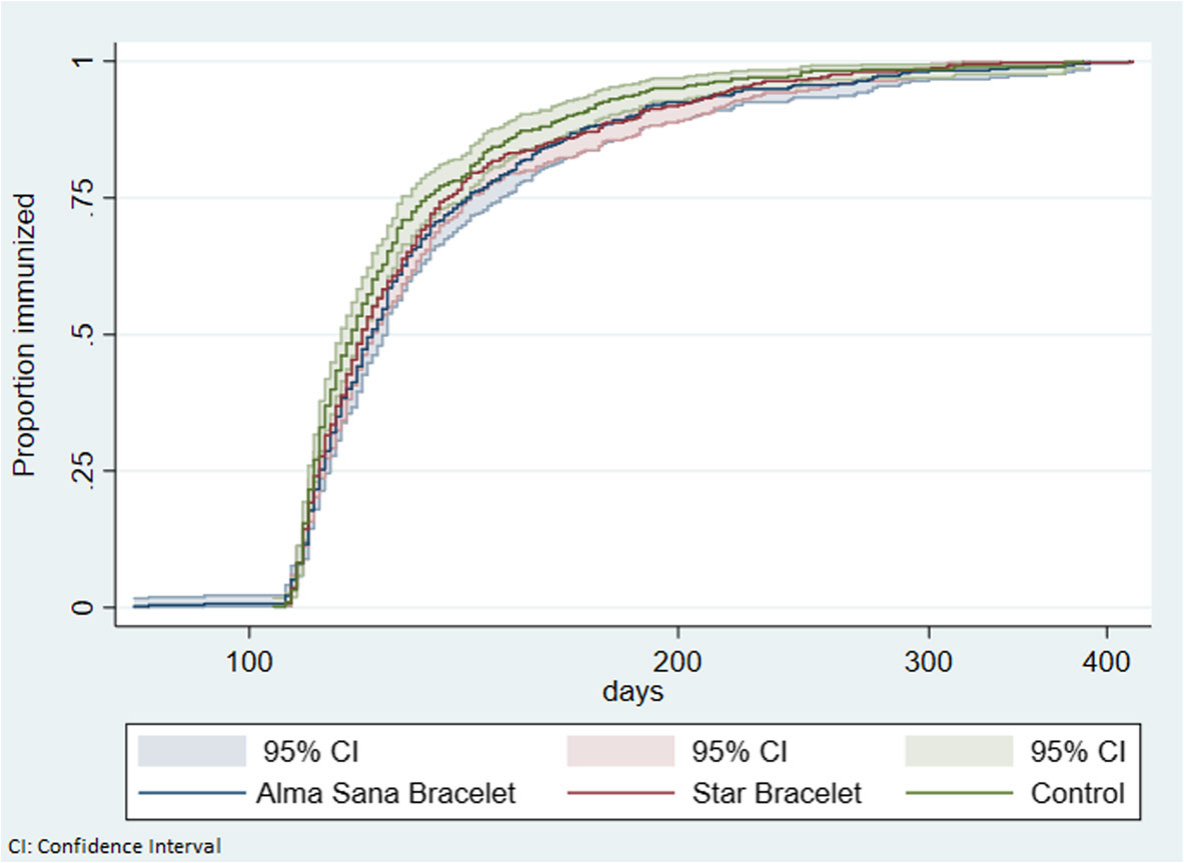
Age at immunization in children for up-to-date Pentavalent-3 completion at 12 months of age by allocation groups

**Fig. 5:**
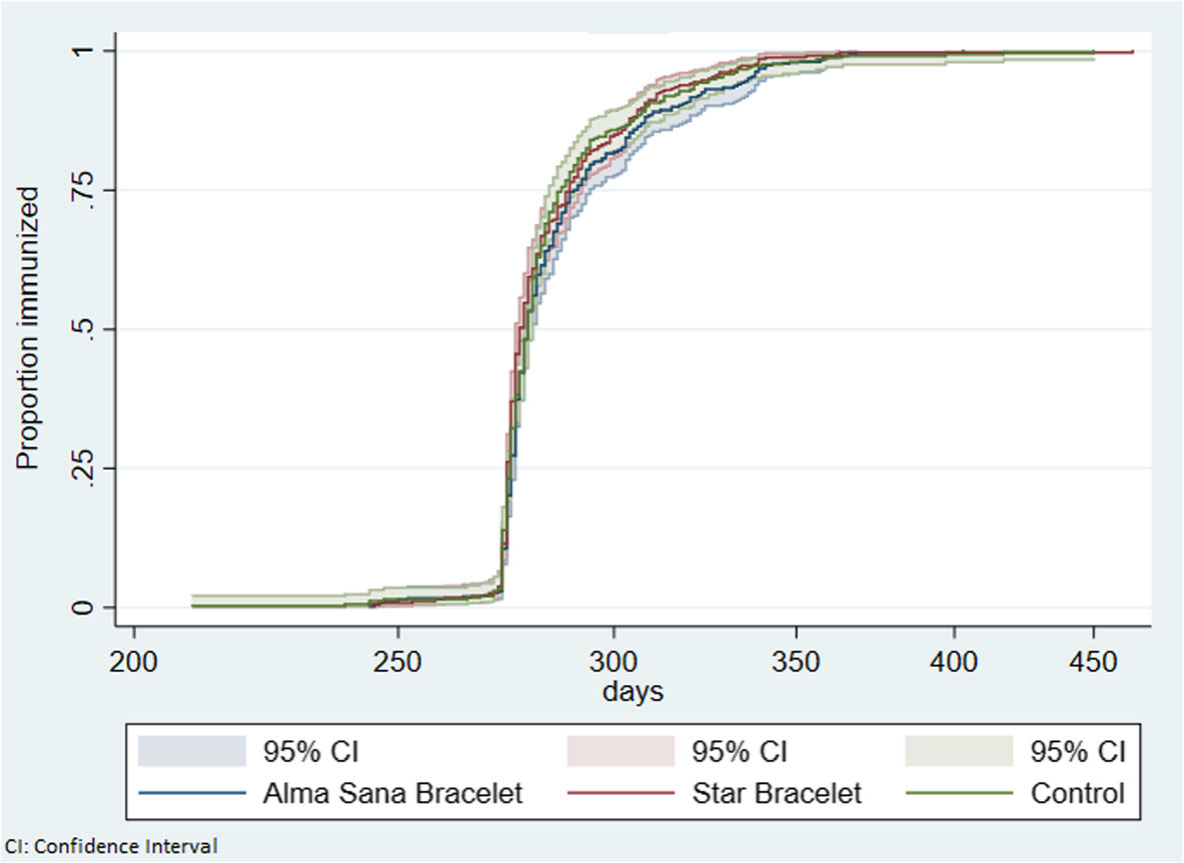
Age at immunization in children for up-to-date Measles-1 vaccine at 12 months of age by allocation groups

The time to immunization for Pentavalent-3 and Measles-1 in the two intervention groups and the control group is shown in Figs. 4 and 5. The figures show that there is no difference in timeliness of Pentavalent-3 or Measles-1 vaccine in either of the intervention groups compared to control group. The median ages of Pentavalent-3 vaccination with survival analysis were 122 days (IQR:112-142) in intervention group A (Alma Sana Bracelet), 120 days (IQR:112-138) in intervention group B (Star Bracelet) and 118 days (IQR: 111-133) in the control group. The median ages of Measles-1 vaccination with survival analysis were 279 days (IQR:275-290) in intervention group A (Alma Sana Bracelet), 277 days (IQR:274-289) in intervention group B (Star Bracelet) and 279 days (IQR: 275-287) in the control group.

In the multivariate analysis, although the outcome variables (coverage rates of Pentavalent-3 and Measles-1) were positive (as shown by RR > 1), neither the Alma Sana bracelet nor the Star bracelet was significantly associated with increase in Pentavalent-3 or Measles-1 vaccination (Table 3). In the multivariate analysis, factors associated with Pentavalent-3 and Measles-1 included father’s education, (RR: 1.01, 95% CI: 1.01-1.02) and (RR: 1.02, 95% CI: 1.01-1.03) respectively, as well as father occupied in a private job compared to skilled labour (RR: 1.11, 95% CI: 1.04-1.18) and (RR: 1.12, 95% CI: 1.02-1.23) respectively. Other factors associated significantly with Pentavalent-3 coverage included employed in an unskilled job (RR: 1.09, 95% CI: 1.01-1.16) compared to employed in skilled labor. We observe a positive and significant association between Measles-1 vaccination and mother’s age (RR: 1.02, 95% CI: 1.01-1.03), whereas there is a negative association between additional number of siblings and Measles-1 vaccination (RR: 0.93, 95% CI: 0.91-0.96).

**Table 3:** Factors associated with Pentavalent-3 and Measles-1 vaccination among study participants

	Factors associated with Penta-3 vaccination among study participants						Factors associated with Measles-1 vaccination among study participants					
	Bivariate Analysis			Multivariate Analysis			Bivariate Analysis			Multivariate Analysis		
	Risk Ratios	95% CI	*p*-Value	Risk Ratios	95% CI	*p*-Value	Risk Ratios	95% CI	*p*-Value	Risk Ratios	95% CI	*p*-Value
Alma Sana Bracelet Group	1.02	0.97-1.07	0.466^*^	1.01	0.96-1.06	0.769	1.05	0.97-1.14	0.235^*^	1.05	0.97-1.13	0.206^*^
Star Bracelet Group	1.02	0.97-1.09	0.420^*^	1.01	0.96-1.06	0.618	1.02	0.94-1.11	0.608^*^	1.03	0.95-1.11	0.474
Gender (female vs. male)	1.00	0.95-1.04	0.854^*^				1.00	0.94 -1.07	0.965^*^			
Paternal education, in years	1.01	1.01-1.02	< 0.0001^**^	1.01	1.01-1.02	< 0.0001^**^	1.03	1.02-1.03	< 0.0001^**^	1.02	1.01-1.03	< 0.0001^**^
Maternal education, in years	1.01	1.01-1.02	< 0.0001^**^				1.02	1.02-1.03	< 0.0001^**^			
Paternal age	1.00	1.00-1.01	0.314^*^				1.00	1.00-1.01	0.122^*^			
Maternal age	1.00	1.00-1.01	0.086^*^				1.01	1.01-1.02	0.012^*^	1.02	1.01-1.03	< 0.0001^**^
Household Income	1.00	1.00-1.00	0.932^*^				1.00	0.99-1.00	0.187^*^			
Fathers Occupation (vs. skilled labour)												
Unskilled labor	1.08	1.01-1.16	0.034*	1.09		0.018*	1.09	0.98-1.22	0.113*	1.10	1.00-1.22	0.060
Retail	1.05	0.98-1.14	0.178*	1.04	1.01-1.16	0.245	1.02	0.91-1.14	0.763*	1.01	0.91-1.13	0.822
Private job	1.14	1.07-1.22	<0.0001**	1.11	0.97-1.12	0.001*	1.21	1.10-1.34	<0.0001**	1.12	1.02-1.23	0.013*
Other	1.10	1.04-1.18	0.003*	1.06	1.04-1.18	0.073	1.21	1.10-1.33	<0.0001**	1.12	1.02-1.24	0.018*
Number of family members	1.00	0.99-1.00	0.975^*^				1.00	0.99-1.01	0.683^*^			
Number of siblings	0.99	0.97-1.00	0.091^*^				0.96	0.93-0.98	< 0.0001^**^	0.93	0.91-0.96	< 0.0001^**^
Number of ANC visits by mother	1.00	1.00-1.01	0.038^*^				1.01	1.01-1.02	< 0.0001^**^			
Place of delivery of child (vs. home)												
Hospital	1.06	0.98-1.17	0.145^*^				1.19	1.03-1.38	0.016^*^			
Who delivered child (vs. doctor)												
Nurse	1.00	1.00-1.01	<0.0001**				1.07	0.90-.023	0.460*			
Midwife	0.81	0.74-0.88	<0.0001**				0.85	0.73-0.99	0.033*			
Other	0.67	0.34-1.17	0.160*				0.70	0.31-1.56	0.383*			
Child is breastfed (no vs. yes)	1.03	0.95-1.12	0.478^*^				1.04	0.91-1.19	0.587^*^			

*p < 0.05

**p < 0.0001

*CI* Confidence Interval, *ANC* Antenatal Care

### Caregiver satisfaction and bracelet visibility

Of the 964 children enrolled in the two intervention groups, 890 (92.3%) were administered the completion form, and the data from their responses is presented in Tables 4 and 5.

**Table 4:** Feedback Regarding Bracelet Utility and Ease of Use from Intervention Group (A & B) Study Participants

Indicators	Intervention A: Adapted Alma Sana Bracelet (*n* = 444)	Intervention B: Star Bracelet (*n* = 446)	Total (*n* = 890)
Method used for vaccine recall, n(%)			
Card only	154 (34.7)	149 (33.4)	303 (34.0)
Bracelet only	7 (1.8)	5 (1.1)	12 (1.3)
Other only	11 (2.5)	9 (2.0)	20 (2.2)
Card and Bracelet	240 (54.1)	252 (56.5)	492 (55.3)
Card and Other	7 (1.6)	7 (1.6)	14 (1.6)
Bracelets and Other	5 (1.1)	6 (1.3)	11 (1.2)
Card and Bracelet and Other	20 (4.5)	18 (4.0)	38 (4.3)
Understood purpose of bracelet (Yes), n(%)	371 (83.6)	382 (85.7)	753 (84.6)
Bracelet reminded regarding upcoming vaccine (Yes), n(%)	313 (70.5)	321 (72.0)	634 (71.2)
Bracelet helped in visiting center on time (Yes), n(%)	230 (51.8)	186 (41.7)	416 (46.7)
Recommend others to use bracelet, n(%)	356 (80.2)	347 (77.8)	703 (79.0)

**Table 5:** Feedback Regarding Bracelet Visibility from Intervention Group (A & B) Study Participants

Indicators	Intervention A: Adapted Alma Sana Bracelet (n = 444)	Intervention B: Star Bracelet (n = 446)	Total (n = 890)
Ever wear bracelet, n(%)	389 (87.6)	389 (87.2)	778 (87.4)
Frequency of wearing bracelet (*n* = 389,389), n(%)			
All the time	56 (14.4)	59 (15.2)	115 (14.8)
Before coming to EPI Center	198 (50.9)	185 (47.6)	383 (49.2)
Sometimes	135 (34.7)	145 (37.3)	280 (36.0)
Duration of wearing bracelet (*n* = 135,145), n(%)			
Rarely (days at a stretch)	27 (20.0)	27 (18.6)	54 (19.3)
Some of the time (weeks at a stretch)	36 (26.7)	42 (29.0)	78 (27.9)
Most of the time (months at a stretch)	72 (53.3)	76 (52.4)	148 (52.8)
Bracelet was within sight when child was not wearing it^a^ (*n* = 388,387), n(%)	309 (79.6)	307 (79.3)	616 (79.5)
Location of bracelet when child was not wearing it^a^, n(%)			
With EPI Card	313 (80.6)	306 (79.1)	619 (79.9)
Bracelet was lost	22 (5.7)	23 (5.9)	45 (5.8)
Others	53 (13.6)	58 (15.0)	111 (14.3)

^a^Denominator based on children who never wore a bracelet or wore bracelet before coming to the center or wore bracelet only sometimes

Almost half of the caregivers (55.3%) (492/890) reported using both the EPI card and bracelet to remind themselves of their child’s vaccination, while a tiny proportion (1.3%) (12/890) reported using the bracelet exclusively (Table 4). Our proxy for measuring overall caregiver satisfaction with the bracelet included whether caregivers understood the purpose of the bracelet (84.6%) (753/890), whether the bracelet reminded them regarding the upcoming vaccine (71.2%) (634/890) and if they would recommend others to use the bracelet (79.0%) (703/890).

A majority of the children (87.4%) (778/890) wore the bracelet at some point during the study, however, out of these, only 14.8% (115/890) wore it all the time whereas almost half (49.2%) (383/890) of the children only wore the bracelet before coming to the EPI center (Table 5). Around 80.0% (616/775) of the caregivers reported that the bracelet was somewhere within their sight when the child was not wearing it, and a similar proportion (80.0%) (619/775) reported that the bracelet was kept with the EPI card when the child was not wearing it. The most commonly cited reasons for not wearing the bracelet at all times were that the caregivers were afraid that the child would lose the bracelet and that the bracelet was not of the appropriate size (results not shown).

## Discussion

We found no significant impact of either the Alma Sana bracelet or the Star bracelet reminders in increasing the up-to-date coverage at 12 months of age or timeliness of Pentavalent-3 or Measles-1 vaccine.

Traditional ‘wearables’ have been used as visual symbols for denoting health indicators since the last several decades. One of the first such tools was a birth control necklace which was developed in collaboration with local Ethiopian women to strengthen awareness regarding the female hormonal cycles [14]. The idea gained traction and was formalized into the ‘Couple/Cycle Bead Method’ that conveyed complex information regarding natural family planning in a simple and visually appealing way. Studies investigating the use of these beaded bracelets found them to be a simple,low-cost, and highly acceptable family planning method [15].

Overtime, the utility of wearables for health has expanded across different disease domains and in recent years, the line between consumer health wearables and medical devices has begun to blur [16]. The upsurge of wearables is mostly concentrated in developed countries, but the concept has gained traction in low and middle-income countries as well where wearable solutions integrating data records such as tattooed bracelets for immunization [17], Near Field Communication (NFC) powered digital pendants [18] and Vaccine Indicator Reminder (VIR) bands [19, 20] are coming to the forefront.

Despite their growing influence, currently, there is limited published literature investigating the impact of these innovative tools. Our study is among the first few attempts to rigorously investigate the impact of these innovations, and our findings are corroborated by a similar study in the region which reported that a Near Field Communication (NFC) powered digital pendant worn as a necklace around the child’s neck did not have any significant impact on DTP-3 vaccination adherence [18].

The underlying appeal and utility of these simple wearables stems from one or more of the following characteristics; their ability to convey complicated information in a simple and easy to comprehend manner, serving as a visual cue and a constant reminder for undertaking the desired actions and lastly, their serving as a social signal among peers.

Our hypothesis that the bracelet would improve immunization uptake was based on the first two characteristics i.e. that the bracelet would serve as a visible and durable reminder as compared to other alternatives, and was easier to understand and interpret for uneducated caregivers.

Contrary to our findings, a study conducted in Sierra Leone using different colored silicone bracelets worn by children as a social signal that the child had completed all required vaccinations saw a 14 percentage point increase in the timely and complete vaccination coverage [21].

It is worth investigating the reasons for the null impact of our study. A key assumption of our intended theory of change was that the bracelets would be a visually evident reminder since they would be visible to the caregivers at all times compared to commonly used alternatives such as immunization cards that are not always within the caregivers’ sight [22]. However, a key finding in our study was the poor compliance of wearing the bracelet; almost half of the study participants only wore the bracelet before coming to the immunization center and only a negligible percentage of children wore the bracelet at all times, despite the study staff reiterating its importance at each follow-up visit.

As part of collecting feedback from caregivers we found out that the most commonly cited reasons for children not wearing the bracelets was their ‘inappropriate size’. Although our findings based on measuring wrist sizes of a sub-sample of children post study showed otherwise, nevertheless this serves as an important guideline to ensure that the bracelets are size-adjustable so that they could comfortably fit the wrist of the child between 0 and 9 months. From a longer term perspective, adherence towards wearing the bracelet also constitutes a behavioral change process which is an important mediator for the observed health outcomes. Health literature elsewhere also highlights that adherence to self-care activities including adopting or refraining from certain behaviors plays an important role in the effectiveness of health care interventions [23]. Moreover, behavior change theories grounded in psychology also emphasize the fact that making health-related behavior changes is a complex process [24] which may lead to an ‘intention-behavior’ gap [25], preventing favorable process outcomes from translating into long term behavior changes. Our findings also provide evidence of the strong reliance on the immunization card being the established immunization recall method and we may also postulate that the short duration of the study did not provide enough time to ‘institutionalize’ the use of the bracelets.

It is worth elaborating more on the favourable feedback from parents. As discussed, a majority of the caregivers in the study found the bracelet to be helpful and expressed a desire to recommend this tool to others. This corresponds to findings from other studies where caregivers in a variety of settings have expressed the need and desire for innovative and novel reminder/recall (R/R) mechanisms [26, 27]. This finding is also in line with results of the study in Udaipur, India where mothers expressed increased satisfaction and acceptability for the novel digital pendant as compared to the traditional immunization reminder mechanism [18]. Our finding, therefore, serves as an important validation of the bracelets in the context of their health-oriented value. In fact, health seeking behaviour in Pakistan specifically and South Asia in general, frequently features faith healers and cultural wearables such as amulets, *ta’wiz*, and pendants that are commonly used for protection [28], which may have led to little resistance from caregivers and facilitated the link between the bracelets and their intended health context.

We, therefore, have a strong reason to believe that our proposed intervention has potential and that certain modifications can allow it to address some of the pervasive issues with conventional R/R mechanisms. Current mechanisms of R/R interventions such as SMS reminders, door-to-door visits, postal reminders, telephone reminders, and community-based counselling have shown mixed results towards improving immunization coverage and timeliness which vary by settings [29]. For instance, the efficacy of Short Message Reminders (SMS) is closely tied to the literacy levels of caregivers as well as the availability of cell phones [30], consistency of phone numbers [31] and network connectivity [13]. Similarly, door to door outreach is expensive and diverts attention away from the quality of immunization service delivery in centers. Additionally, factors such as burden on existing human resource, uncertainty about who should implement reminder services, high costs and lack of high-quality immunization records have all been cited as barriers towards the adoption of more technology dependent reminder services [32] .

Our study has certain limitations; as a result of limited time and resources, we could only follow up our study participants up till the administration of Measles-1 vaccine (recommended age 9 months) which constitutes the second last dose of the routine immunization schedule. It is difficult to predict whether we would observe a similar impact of our intervention for the Measles-2 coverage rate (recommended age 15 months) where the incidence of drop out is highest [4, 33] and retention of immunization cards is also lowest [34] . Additionally, our study only enrolled children who showed up at clinics for immunization and not those who were not vaccinating in the first place. We do acknowledge that the bracelets may have had an impact on never vaccinated children in the community due to positive externalities. However, it was beyond the scope of this study to evaluate this indirect impact and hence enrolment was only confined within the clinic setting.

Our findings also point to some critical implications for future work and for similar novel innovations targeted towards improving caregiver adherence to the routine immunization schedule. Given the widely reported positive impact of community-based educational interventions on enhancing immunization coverage [35] it is worth suggesting that any similar novel innovation or tool is accompanied by community mobilization and engagement to emphasize the importance and utility of the innovation. This is also consistent with the literature on adherence and compliance of health-related behavior whereby good communication strategies, counseling, and knowledge are important predictors of adherence [36]. Furthermore, we believe that the delivery of the bracelets through trusted vaccinators or community health workers as opposed to ‘distant’ study staff would give it more legitimacy and enable better compliance towards wearing the bracelet. This is in line with other findings where parental perceptions towards R/R services shows that delivery of R/R services by the government or through the established health network was preferred by the caregivers [37]. Furthermore, areas that warrant further research include: evaluating the impact of the bracelet in a purely rural community where literacy rates are much lower, introducing the bracelets even earlier on (since immunization coverage of children enrolled at BCG was higher than for those enrolled at Pentavalent-1) and considering designing bracelets for mothers to be provided at the time of antenatal care visits as studies have shown a positive association between mothers antenatal care visits and subsequent uptake of child immunizations [38, 39].

## Conclusion

We did not observe any significant impact of the two types of bracelets on improved immunization coverage and timeliness in the treatment groups. Our findings add to the existing literature on innovative, low cost reminders for health and provide impetus for future research on how we can enhance the practical implementation of these bracelets. These include accompanying introduction of the bracelet with a community mobilization component, making the bracelets size-adjustable, and introducing them even earlier on in the immunization cycle. We make several suggestions for the future use of the bracelets and other similar low cost reminder interventions aimed at improving immunization coverage and timeliness across similar settings.

## Supplementary information

**Additional file 1:Figure 1.** Schedule of enrolment, interventions, and assessments for children enrolled in the study.

## Data Availability

The datasets used and/or analyzed during the current study are available from the corresponding author on reasonable request.

## Abbreviations

BCG: Bacille Calmette Guérin
CI: Confidence Interval
DTP-3: Diphtheria-tetanus-pertussis
EPI: Expanded Programme on Immunization
Gavi: Gavi, The Vaccine Alliance
IQR: Inter Quartile Range
KM: Kamplan Meir
NFC: Near Field Communication
PCV: Pneumococcal Conjugate Vaccine
RR: Risk Ratio
R/R: Reminder/Recall
SD: Standard Deviation
SMS: Short Message Service
USD: United States Dollar
VIR: Vaccine Indicator Reminder
