# Supplementary material for: Effect of vaccine reminder and tracker bracelets on routine childhood immunization coverage and timeliness in urban Pakistan (2017-18): a randomized controlled trial
Source: BMC Public Health. 2020 Jul 11;20:1086. doi: 10.1186/s12889-020-09088-4 (PMC7353686; doi:10.1186/s12889-020-09088-4)
Supplement: Supplementary file 1 — Additional file 1:Figure 1. Schedule of enrolment, interventions, and assessments for children enrolled in the study. [file 12889_2020_9088_MOESM1_ESM.doc]

Supplementary Figure 1: Schedule of enrolment, interventions, and assessments for children enrolled in the study.

|  | **STUDY PERIOD** | | | | | | |
| --- | --- | --- | --- | --- | --- | --- | --- |
|  | **Enrolment** | **Allocation** | **Post-allocation follow up** | | | | **Completion** |
| **TIMEPOINT** | **0-12w** | **0-12w** | ***6w*** | ***10w*** | ***14w*** | ***36w*** | ***36-48w*** |
| **ENROLMENT:** |  |  |  |  |  |  |  |
| **Eligibility screen** | X |  |  |  |  |  |  |
| **Informed consent** | X |  |  |  |  |  |  |
| **Baseline Data Collection** | X |  |  |  |  |  |  |
| **Allocation/Randomization** |  | X |  |  |  |  |  |
| **INTERVENTIONS:** |  |  |  |  |  |  |  |
| ***Alma Sana Bracelet*** |  |  |  |  |  |  |  |
| ***Star Bracelet*** |  |  |  |  |  |  |  |
| **ASSESSMENTS:** |  |  |  |  |  |  |  |
| ***Demographic data*** | X |  |  |  |  |  |  |
| ***Immunization history of the child*** |  |  | X | X | X | X | X |
| ***Compliance Data on wearing bracelet and feedback on bracelet*** |  |  |  |  |  | X | X |

*The time periods denote the age of the children enrolled in the study.

For children enrolled at the BCG vaccine, their first follow-up visit would be at 6 weeks of age.

For children enrolled at the Pentavalent vaccine, their first follow-up visit would be at 10 weeks of age.
